# Neuropsychological profile of hearing-impaired patients and the effect of hearing aid on cognitive functions: an exploratory study

**DOI:** 10.1038/s41598-021-88487-y

**Published:** 2021-04-30

**Authors:** Sofia Cuoco, Arianna Cappiello, Alfonso Scarpa, Donato Troisi, Maria Autuori, Sara Ponticorvo, Claudia Cassandro, Renzo Manara, Fabrizio Esposito, Gabriella Santangelo, Paolo Barone, Ettore Cassandro, Maria Teresa Pellecchia

**Affiliations:** 1 Neuroscience Section, Department of Medicine, Surgery and Dentistry, “Scuola Medica Salernitana“, University of Salerno, Salerno, Italy; 2 Department of Medicine, Surgery and Dentistry, “Scuola Medica Salernitana”, University of Salerno, Salerno, Italy; 3 Department of Surgical Sciences, University of Turin, Turin, Italy; 4 Department of Psychology, University of Campania Luigi Vanvitelli, Caserta, Italy; 5 Center for Neurodegenerative Diseases (CEMAND), University of Salerno, 84131 Salerno, Italy

**Keywords:** Cognitive ageing, Cognitive neuroscience, Neuroscience, Risk factors

## Abstract

Few studies have investigated the neuropsychological profile of Hearing Loss (HL) subjects and the effects of hearing-aid on cognitive decline. We investigated the neuropsychological profile of HL patients at baseline and compared the neuropsychological profiles of patients with and without hearing-aid at 6 month. Fifty-six HL patients and 40 healthy subjects (HC) underwent neuropsychological and behavioral examination and were compared at baseline. Changes at follow-up were compared between HL patients with (N = 25) and without (N = 31) hearing-aids. At baseline, significant differences between HL and HC were found in MOCA test, Raven's Coloured Progressive Matrices (CPM) and SF-36. Among mild-HL patients, patients with hearing-aid significantly improved on the Clock Drawing Test (CDT) as compared to patients without hearing-aid. Our findings indicate that hearing loss is associated with both a reduced efficiency of the global cognitive state and a worse quality of life as compared to HC, supporting the association between HL and cognitive impairment. Moreover, only patients with mild-HL shows some cognitive improvement after using hearing-aid, suggesting that rehabilitative strategies may be more effective to delay cognitive decline in such patients. However, we cannot exclude that hearing-aids may affect cognitive decline in more severe-HL, but a longer follow-up is needed.

## Introduction

Hearing loss (HL) is characterized by poor speech perception and understanding, especially in noisy settings that involves 25–30% of individuals between 65 and 74 years^1^. HL causes difficulties in both professional and social life, as well as stigmatization. It is estimated that, by 2025, 900 million people throughout the world will be hearing-impaired^2,3^.

The relationship between auditory and cognitive functioning in the elderly has been the subject of many studies and, although many cross-sectional or longitudinal cohort studies found an association between hearing loss and cognitive decline or dementia^4,5^, conflicting results have been reported^6^. In fact, current evidence cannot confirm a clear causal relationship between HL and cognition, since it’s supposed that auditory deafferentation may be a determinant of cognitive decline, but it’s also possible that HL and cognitive declines hare common underlying pathological processes in older age^6^. Increasing evidence suggests that hearing loss is associated with a wide range of health issues, higher disease burden, and increased risk for hospitalization^6,7^.

On the other hand, recent studies showed that reduced auditory input due to a hearing impairment is also associated with greater declines in cognitive function in older adults^8^. In particular, HL patients showed a greater risk of developing dementia and a global decrease of cognitive performances^9,10^. Attention and processing speed are the most affected functions in HL patients, that appear to need more resources to complete the tasks^11^ but also auditory-verbal memory is affected in HL^12^. Various hypotheses have been proposed to explain cognitive decline associated with hearing impairment. HL and cognitive decline may have a common neuropathological origin but it is also possible that hearing loss alone could promote deficits in different areas or interact with other risk factors accelerating cognitive decline^8^. Usually, HL seems to speed up cognitive decline through the combination of social and sensory isolation and through an increased cognitive load^13^. Indeed, peripheral HL decreases local cerebral blood flow in primary auditory cortex^14^ and it is associated with decreased volumes in the auditory cortex, temporal lobe and whole brain, and also with a functional reorganization of the auditory cortical networks, consistent with more demanding listening and a reduction of cognitive reserve^13^. Furthermore, a reduction of perfusion in primary auditory cortex has been found by ASL-MRI in patients affected by HL in the early stages, suggesting that there is a relationship between HL and cortical activation/metabolism, even if no atrophy was found^14^.

Comprehensive hearing rehabilitative interventions, that incorporate the use of sensory aids (hearing aids) and rehabilitative counseling to maximize the audibility of speech signals and reduce the cognitive load, provide increased auditory stimulation, and promote social engagement. However, only few studies have investigated the effects of hearing rehabilitative interventions on cognitive decline in HL patients. Specifically, the tests used so far to measure cognitive deficits in HL patients are very heterogeneous and only recently more complete neuropsychological batteries have been used on large samples^11,15^. Differences in administered tests, sample selection variables and, protocols for the use of aids also led to heterogeneous results^3,11,15^.

Therefore, the purposes of the present study were (1) to comprehensively investigate the neuropsychological profile of patients with HL at baseline (T_0_) and (2) to compare the neuropsychological profiles of patients with and without hearing aid at 6 month-FU (T_1_).

## Patients and methods

### Patients

Fifty-six (M = 35, F = 21) consecutive patients diagnosed with HL (mean + SD PTA threshold: 51.18 + 13.38 dB), and 40 healthy subjects (HC), matched for age and education, were recruited at the S. Giovanni di Dio and Ruggi D’Aragona Hospital, Salerno, Italy, between September 2018 and November 2019.

Inclusion criteria were the following: patients older than 55 years without dementia, which was evaluated by Mini-Mental State Examination (MMSE) (correct score < 23.8).

Exclusion criteria of recruitment in the study were the following: (i) stroke or other neurological disorders; (ii) global cognitive impairment; (iii) major psychiatric disorders; (iv) other causes of hearing loss different from presbycusis (e.g., conductive hearing loss); (v) patients using hearing aids and (vi) other causes of significant disability.

All the audiological evaluations were carried out by an experienced audiologist in a double wall sound proof room. Air conduction pure tone audiometric hearing thresholds were evaluated at 0.125, 0.25, 0.5, 1, 2, 3, 4, 6 and 8 kHz for each subject in both ears using a clinical audiometer (AC40, Interacoustics R). In addition, in order to rule out conductive hearing loss, bone conduction thresholds were measured at 0.25, 0.5, 1, 2, 3 and 4 kHz. All audiometric data presented in the present work refer to air conduction thresholds. The Pure Tone Average (PTA) was calculated ad mean of pure tone thresholds at 0.5, 1, 2 and 4 kHz for each subject in both ears. Hearing impairment was ranked based on PTA as mild, moderate, severe, or profound as follows: mild loss, 26–40 dB; moderate loss, 41–70 dB; and severe loss, 71–90 dB, profound, 90+^12^. Given the reduced sample size of the PTA-groups, also divided between with and without aids, we have combined 3 patients with PTA-profound (G.D. = 91.25, C.S. = 96.25, V.S. = 93.75) with patients with PTA-severe.

Twenty-five patients were compliant and accepted to use hearing aids, as prescribed by the audiologists (otolaryngologist), whereas the remaining 31 patients did not accept to wear hearing aids. Neuropsychological and emotional-behavioral examination was repeated in patients with and without hearing aids at 6-month follow-up.

We choose a battery of tests aimed to reduce confounding factors possibly overestimating the strength of the association between HL and cognitive deficit^6^.

The neuropsychological battery included the Montreal Cognitive Assessment (MOCA) to investigate the global cognitive state. In order to analyses the other cognitive functions we used the Digit Cancellation Test (DCT) and the Trail Making Test-part A (TMT-A), The Rey’s auditory 15-word learning test to assess verbal immediate recall and verbal delayed recall (15-RAWLT),the Rey-Osterrieth Complex Figure Test memory (ROCF memory) ,and copy (ROCF copy) and the Clock Drawing Test (CDT). Finally, we used the Raven's Coloured Progressive Matrices (CPM) as screening and executive function test, since, as suggested by Spreen and Strauss^16^, it is recommended for subjects with deafness. Specifically, the CPM has classically been used to measure global cognitive performance (in terms of intelligence)^17^, mental age^18^, intellectual performance^19^ and non-verbal intelligence^20^, or to measure visuo-perceptual and visuo-spatial functions^21^. Moreover, studies of brain activity or structure suggest frontal involvement in CPM performance^20^.

In order to investigate the quality of life and the presence of emotional disorders, such as depression and apathy, Short Form 36 (SF-36), Beck Depression Inventory II (BDI-II; cut-off > 12) and Dimensional Apathy Scale (DAS) were administered.

The study was conducted in accordance with the Declaration of Helsinki. All people provided informed consent before being included in the study. The protocol was approved by the competent local committee in charge of guaranteeing the protection of the subjects, such as Campania-South Ethics Committee.

### Statistical analysis

Normality test was conducted for demographic, neuropsychological and behavioral variables.

An exploratory factor analysis, with Varimax’ rotation statistical technique, was conducted in order to combine the constructs related to the proposed neuropsychological battery and we conducted exploratory post-hoc power analysis for each factor to evaluate the sample size.

Patients were compared with HC for demographic and neuropsychological data at baseline by Mann–Whitney U test, corrected for multiple comparisons.

We assessed the relationship between depression and quality of life (QoL) at baseline in HL patients by Spearman’s correlation analysis. Changes in neuropsychological and behavioral scores between T_1_ and T_0_ were calculated (Delta = T_1_ – T_0_) and compared between patients with and without hearing aids using Mann–Whitney's U test. Significant results obtained from the exploratory analysis were then corrected for multiple comparisons.

We also used the Mann–Whitney’s U test to compare neuropsychological and behavioral parameters at baseline and changes at follow up between HL patients with and without hearing aid divided according to the level of hearing impairment.

### Statement

All coauthors agreed with the contents of manuscript. The submission is not under review at any other publication. The study with human subjects was conducted in accordance with the Declaration of Helsinki. All people provided informed consent before being included in the study. The protocol was approved by the competent local committee in charge of guaranteeing the protection of the subjects, such as Campania-South Ethics Committee.

## Results

### Comparison between healthy subjects and HL patients

According to exploratory factor analysis, there were three latent factors underlying the neuropsychological tests. The first factor explained the 46% of the cumulative variance, the first and second factors explained 61% of the cumulative variance and the three factors together explained 72% of the cumulative variance. We defined the first factor “executive/ attentional factor”, the second one as “memory factor” and the third one as “visuo-spatial factor” (see Supplemental Digital Content-Table S1 for the variables’ distribution and post-hoc power analysis)”.

The patients and HC did not differ for age and education (p = 0.405; p = 0.749) (Table 1).

**Table 1:** Demographic, neuropsychological and behavioral features in HL patients and healthy controls.

	HL patients (N = 56) Median (IQR)	HC (N = 40) Median (IQR)	P
Age, years	64.26 ± 7.97	63.22 ± 6.33	0.405
Age of education	10.92 ± 4.87	11.27 ± 4.69	0.749
CPM	25.60 ± 6.05^a^	29.94 ± 6.34^a^	**0.001**
MOCA-total	21.05 ± 4.6^a^	23.67 ± 3.45^a^	**0.004**
15-RAWTL-immediate recall	33.26 ± 11.11	38.97 ± 10.03	**0.010**
15- RAWTL-delayed recall	6.41 ± 2.83	8.00 ± 3.05	**0.010**
ROCF memory	13.21 ± 6.55	13.77 ± 6.21	0.652
ROCF copy	29.49 ± 7.30	32.10 ± 4.55	0.170
CDT	11.01 ± 13.54	9.55 ± 0.98	0.406
TMT-A	48.92 ± 27.04	48.57 ± 14.88	0.298
Odct	49.07 ± 8.30	50.56 ± 7.01	0.493
BDI-II	8.83 ± 8.89	4.84 ± 4.73	*0.064*
DAS-total	21.21 ± 9.77	18.69 ± 7.96	0.231
DAS-executivel	6.28 ± 4.72	5.00 ± 4.58	0.174
DAS-emoziona	7.67 ± 4.08	6.73 ± 3.56	0.275
DAS-initiation	7.26 ± 4.57	6.96 ± 4.34	0.940
Sf-36 physical function	74.73 ± 25.46	79.20 ± 29.70	0.190
Sf-36 role limitations due to physical problems	55.03 ± 40.76	73.95 ± 37.93	**0.043**
Sf-36 bodily pain	52.10 ± 31.57^a^	73.95 ± 23.25^a^	**0.004**
Sf-36 general health perception	47.67 ± 23.48^a^	65.41 ± 17.49^a^	**0.001**
Sf-36 emotional wellbeing	49.55 ± 23.10^a^	66.45 ± 14.48^a^	**0.001**
Sf-36 social functioning	63.75 ± 28.93	84.62 ± 19.25	**0.005**
Sf-36 role limitations due to emotional problems	59.96 ± 42.36	84.62 ± 31.12	**0.011**
Sf-36 general mental health perception	55.74 ± 23.82^a^	74.91 ± 13.12^a^	** < 0.001**

Statistically significant differences are indicated in bold. The trend towards statistical significance is indicated in italics.

15-RAWLT Rey’s auditory 15-word learning test- Verbal immediate recall; 15-RAWLT, Rey’s auditory 15-word learning test- Verbal delayed recall; BDI-II; Beck Depression Inventory II;CDT, Clock Drawing Test; CPM, Raven's Coloured Progressive Matrices; DAS, Dimensional Apathy Scale; HC, Healthy controls; HL, hearing loss; IQR, Interquartile ranges; MOCA, Montreal Cognitive Assessment; N, simple size; oDCT, Digit Cancellation Test; ROCF, Rey-Osterrieth Complex Figure Test; SF 36, Short form 36; TMT-A, Trail Making Test Part A.

^a^HL patients vs HC corrected for p ≤ 0.004.

A significant difference was found between the two groups in the following tests: MOCA-total score (p = 0.004), CPM (p = 0.001), SF-36 bodily pain (p = 0.004), SF-36 general health perception (p = 0.001), SF-36 Emotional wellbeing (p = 0.001) and SF-36 mental health (p < 0.001), with all average values resulting higher in HC compared to patients. Therefore, HL had worse performances on cognitive tests and a worse quality of life (Table 1). After the exploratory analysis, depression was more present in HL than HC (BDI-II, p = 0.064), but this difference did not reach statistical significant (Table 1). Although after correction for multiple comparison also the trend detected on BDI-II scale was no significance, we found that 26% of HL patients showed a BDI-II score higher than 12 as compared to 8% of HC (p = 0.077). Significant negative correlations were found between BDI-II and eight domains of the SF-36: physical function (rho = − 0.362, p = 0.007), role limitations due to physical problems (rho = − 0.490, p < 0.001), social functioning (rho = − 0.426, p = 0.001), bodily pain (rho = − 0.515, p < 0.001), general mental health perception (rho = − 0.492, p < 0.001), role limitations due to emotional problems (rho = − 0.0438, p = 0.001), emotional wellbeing (rho = − 0.492, p < 0.001), and general health perception (rho = − 0.525, p < 0.001).

### Comparison of patients with and without hearing aids

The patients not accepting hearing aids did not differ from patients accepting hearing aids for age, education and cognitive tests. At baseline, patients not accepting hearing aids showed higher scores only on a subcomponent of DAS as compared to patients accepting hearing aids, as analyzed by Mann–Whitney test and corrected for multiple comparisons (p ≤ 0.025).

After the exploratory analysis, significantly different changes (T1 − T0) were found between patients with and without hearing aid on the CPM (p = 0.002), the ROCF (p = 0.032), the DAS-Total (p = 0.016) and DAS-emotional (p = 0.048). In particular, patients with hearing aid improved on the ROCF and DAS and worsened on the CPM, as compared to patients without hearing aid, but after correcting for multiple comparisons only CPM differences were still significant between groups (Table 2).

**Table 2:** Delta (T_1_ – T_0_) values for neuropsychological and behavioral variables in HL patients with and without hearing aids.

	HL patients with aids (N = 25) Median (IQR)	HL patients without aids (N = 31) Median (IQR)	P
Age, years	62.64 ± 7.89	65.58 ± 7.93	0.168
Age of education	10.80 ± 4.83	11.00 ± 4.98	0.822
CPM	− 2.04 ± 6.42^a^	1.58 ± 3.42^a^	**0.002**
MOCA-total	− 0.08 ± 2.98	0.06 ± 3.39	0.941
15-RAWTL-immediate recall	1.80 ± 8.39	3.35 ± 6.87	0.525
15-RAWTL-delayed recall	1.20 ± 1.82	0.80 ± 2.54	0.893
ROCF memory	4.54 ± 8.27	0.22 ± 6.12	**0.032**
ROCF copy	− 0.62 ± 3.76	− 0.50 ± 3.70	0.698
CDT	0.00 ± 1.00	− 3.16 ± 18.25	0.962
TMT-A	− 1.64 ± 18.11	3.74 ± 16.54	0.158
oDCT	1.16 ± 12.53	− 1.22 ± 6.87	0.980
BDI-II	− 2.91 ± 9.13	− 1.90 ± 9.61	0.518
DAS-total	14.29 ± 19.32	0.31 ± 25.98	**0.016**
DAS-executive	0.33 ± 5.08	− 0.44 ± 4.21	0.222
DAS-emotional	− 1.37 ± 3.93	1.82 ± 5.55	**0.048**
DAS-initiation	0.95 ± 4.81	0.72 ± 5.33	0.693
Sf-36 physical function	− 3.80 ± 23.64	− 5.96 ± 33.07	0.712
Sf-36 role limitations due to physical problems	8.08 ± 40.33	− 2.41 ± 50.56	0.636
Sf-36 bodily pain	9.52 ± 32.14	− 4.22 ± 35.82	0.077
Sf-36 general health perception	− 3.32 ± 22.18	− 2.25 ± 18.44	0.954
Sf-36 emotional wellbeing	− 1.4 ± 27.21	1.61 ± 21.77	0.690
Sf-36 social functioning	4.84 ± 33.82	− 2.77 ± 32.16	0.310
Sf-36 role limitations due to emotional problems	6.80 ± 50.02	0.09 ± 53.06	0.647
Sf-36 general mental health perception	− 2.00 ± 30.73	2.32 ± 22.71	0.707

Statistically significant differences are indicated in bold.

15-RAWLT Rey’sauditory 15-word learning test- Verbal immediate recall; 15-RAWLT, Rey’sauditory 15-word learning test- Verbal delayed recall; BDI-II, Beck Depression Inventory II; CDT, Clock Drawing Test; CPM, Raven's Coloured Progressive Matrices; DAS, DimensionalApathy Scale; HL, hearing loss; IQR, Interquartile ranges; MOCA, Montreal Cognitive Assessment; N, simplesize;oDCT, DigitCancellation Test; ROCF, Rey-OsterriethComplex Figure Test;; SF 36, Short form 36; T_0_ = base-line; T_1_ = Follow up; TMT-A, TrailMaking Test Part A.

^a^HL patients with aids vs HL patients without aids, corrected for p ≤ 0.0125.

No significant differences in cognitive or behavioral parameters were found at baseline among patients with mild, moderate or severe- profound HL. Among patients with mild HL, patients with hearing aid significantly improved on the CDT (p = 0.039) as compared to patients without hearing aid. Among patients with moderate HL, patients with hearing aid showed a trend towards a significant improvement on ROCF memory (p = 0.065), but worsened on CPM as compared to patients without hearing aid (p = 0.038). Among patients with severe-profound HL, patients with hearing aid significantly improved on DAS (p = 0.032), while worsening on CPM (p = 0.027) as compared to patients without hearing aid (Table 3).

**Table 3:** Significant differences in delta (T1 − T0) values for neuropsychological and behavioral variables in HL patients with and without hearing aids divided according to the severity of HL .

	HL patients with aids (N = 4)	HL patients without aids (N = 6)	P
**Mild HL**			
CDT	1	− 1.66	**0.039**

	HL patients with aids (N = 6)	HL patients without aids (N = 5)	P
**Moderate HL**			
CPM	− 1	1.05	**0.038**
ROCF memory	6.11	− 0.15	0.065

	HL patients with aids (N = 13)	HL patients without aids (N = 20)	P
**Severe-profound HL**			
CPM	− 5.8	3.2	**0.027**
DAS-total	25.33	− 12.2	**0.032**

Statistically significant differences are indicated in bold.

CDT, Clock Drawing Test; CPM, Raven's Coloured Progressive Matrices; DAS, Dimensional Apathy Scale; HL, hearing loss; N, simple size; PTA, pure tone audiometric; ROCF memory, Rey-Osterrieth Complex Figure Test; T0, base-line; T1, Follow up.

## Discussion

### Comparison between healthy subjects and HL patients

Epidemiological evidence suggests a relationship between HL and cognitive functions in adults over the age of 60, but there are still few studies on cognitive profile of HL patients^11,15^ and despite a huge research effort, elucidation of the causal relationship between auditory and cognitive decline has not yet reached a consensus^22^. In fact, such association could also be due to shared pathological mechanisms, still unclear^23^, since hearing loss is supposed to affect different domains of aging through different mechanistic pathways not mutually exclusive^24^.

Our study aimed to comprehensively evaluate the cognitive and behavioral features of subjects with HL compared with HC and to assess the effect of hearing aids, used for 6 months, on cognitive and behavioral tests.

Since the comprehension of verbal instructions during cognitive assessment is highly dependent on hearing, the risk of overestimation of the level of cognitive impairment is high in hearing impaired individuals^25^. For this reason, we selected, as far as possible, cognitive tests with reduced verbal load. One of the most reliable tools is CPM, because the delivery is brief and the requested response is not verbal. Moreover, CPM is a measure of the global cognitive state but also of executive functions^17,19,20^. We found that all the neuropsychological tests used are included in three macro-constructs, that we defined as executive/attentional, memory and visuo-spatial factors. Despite the MOCA is a global screening test, it was included in the executive/attentive factor because it relies on frontal executive and attention tasks much more than the MMSE^26^. We included the ROCF- memory task into the executive/attention factor given the weight of planning skills in this task and the CDT in visuo-spatial factor given the weight of visual-constructive skills (see Supplemental Digital Content-Table S2). The low or moderate power of the three factors may be linked with the small sample and/or the difficulty in selecting adequate tests for HL patients, however, considering the individual neuropsychological tests, the power of the global cognitive state tests (CPM and MOCA) was good as compared to other tests.

In our study HL patients, compared with HC, showed worse performances on test assessing the cognitive state, such as the MOCA test and the CPM. Our results are partially in line with previous studies that reported a relationship between hearing impairment and multiple domain cognitive deficits. Indeed, we recognize that MOCA test may be affected by hearing loss^27^, but the MOCA version adapted for people with hearing loss was published only after we started study recruitment^28^. Nevertheless, the standard MOCA test is one of the most complete and reliable screening tests^28^ and we believe that our MOCA results are reliable as they reflect findings from CPM, that is another measure of the global cognitive state, also recommended for patients with HL^17–19,21^.

More specifically, CPM results are also in line with previous studies that reported a greater involvement of executive functions in hearing-impaired patients^11,29,30^. Although CPM was recommended for HL, it was not used in most of the previous studies, while in the study by Gugliemi et al.^31^ it did not show significant differences between HL patients and HC subjects. The difference observed in CPM between our study and the previous one^31^ could be due to an older and more educated HL sample as compared to ours (see Supplemental Digital Content-Table S3). Anyway, beyond the performance reported at a single test, it is recognized that executive functions of HL subjects are negatively affected by the increase in workload, and a slowdown in the speed of information processing could explain our results^32^. Indeed, a recent study suggested that impaired cochlear amplification mechanism could lead to cognitive deficits; in fact, HL patients with greater impaired cochlear amplification mechanism also had a greater cerebral atrophy in the cingulate and parahippocampal cortex^30^. The inefficient modulation of salience network, formed by anterior cingulate cortex, anterior insula, subcortical and limbic structures that are also involved in auditory perception, could contribute to cognitive and emotional deficits in HL patients^33^. Our study showed significant differences on tests that evaluate global cognitive status but not specific cognitive functions. We believe that this may partially depend on the limited size of our sample, in part on the instruments used, since specific neuropsychological tasks are not yet available for HL patients. Nevertheless, we hypothesize that more global tools, such as MOCA or CPM, may be more responsive to change in this population as compared to specific domain tests due to the compensatory changes in cortical resource allocation reported in patients with HL since the early stages^34^.

As regards the domain of memory, we did not find that HL patients performed worse than HC on tasks assessing learning and recall of verbal material, correcting our data for multiple comparisons. But in the our exploratory analysis a difference in the memory domain is reported and which is partially in line with previous studies^12,31,35^. In fact, just recently, Guglielmi et al.^31^ found that 40 HL subjects did not have the same performance as healthy subjects in tests investigating short-term memory but showed different performances in long-term. Shahidipour et al.^12^ reported a strong association between sensory deficit and performance on tests investigating verbal auditory memory in 23 HL patients compared with 24 HC. They explained their results according to the “effort-fullness” hypothesis, suggesting that the extra efforts required to achieve perceptual success in a hearing impaired listener may affect processing resources usually available for encoding the speech content in memory^12^. We suggest that our results partly support this hypothesis but also the caution in interpreting the results related to verbal memory tests which inevitably suffer greatly from the hearing problem. Analyzing the literature, we found that the memory domain was often studied and reported as deficient in patients with HL studied by means of heterogeneous verbal memory tests but, in relation to the hearing bias, we did not believe that these were the optimal tests to compare the cognitive functions of HL patients with healthy ones (see Supplementary digital content—Tables S2 and S3).

Lin et al.^35^ assessed 605 participants, 433 of which with normal hearing, 128 with mild HL, 43 with moderate HL and one with severe- profound HL, by means of Digit Symbol Substitution Test (DSST), used to analyze attention or global cognitive status, and found that greater hearing loss was significantly associated with lower DSST scores. As for attention, we used Digit Cancellation Test (DCT) and the Trail Making Test-part A (TMT-A) to measure task-switching and information processing speed and selective attention but we found no significant differences; this may depend on the size of the sample and the type of specific function studied.

Finally, in the previous literature memory and executive constructs were usually affected in HL patients (see Supplemental Digital Content—Table S3), but there were heterogeneous results possibility depending on the tests selected to represent a domain, but also on the sample and administration methods. In our sample, the tests used for the same domain did not necessarily show similar differences between the groups and this can be justified by the fact that although a test is representative of the same domain it can never reflect perfectly the same functions of another test. Therefore, in HL patients we suggest to focus more on tests such as MOCA or CPM, which are an expression of global cognitive state.

Since the auditory system features an intense efferent and afferent traffic due to mutual interactions between cortical, brain stem and peripheral pathways, the impact of hearing loss on cognitive decline should be considered as dependent on both central and peripheral factors^13^. Also the reduction of social interactions, due to a decreased understanding of conversations, could further accelerate cognitive decline^36^. With regard to the effects of hearing impairment on brain structure/function, Peelle et al.^37^ showed that poorer hearing is associated with a reduced language-driven activity in primary auditory pathways and an increased compensatory language-driven activity in pre-frontal cortical areas^37^. As for the behavioral-emotional variables, as expected, the HL patients showed a worse quality of life than the HC; furthermore, although there is no significant difference in BDI-II, when assessing the cognitive and somatic-affective symptoms of depression, there was a higher raw score for patients than HC. Specifically, our HL patients showed lower scores in the domains of physical and emotional quality of life (QoL), such as pain, emotional wellbeing, general health, and mental health, as previously reported^38,39^. Polku et al.^38^ found that only perceived hearing difficulty factors, as assessed using a 16-item questionnaire sent by post, and not objective HL severity as assessed by pure tone audiometry, were significantly associated with lower scores on total and all QoL domains, assessed by the short version of the 26-item World Health Organization Quality of Life Assessment. Hyams et al.^39^ evaluated 30 HC, 33 hearing impaired subjects without hearing aids, and 37 hearing impaired subjects with hearing aids using the Short Form-36 and found that subjects without hearing aids had significantly lower QoL than those without hearing aids. hearing impaired with hearing aids and HC. In the elderly population with HL, up to 20% of subjects report a clinically relevant level of depressive symptoms that would require treatment^40^. Both cross-sectional and longitudinal studies indicate that HL is related to the increase in depressive symptoms, although the strength of the association varies between studies. Several methodological differences, such as age at enrollment, severity of HL, and the scales used for assessing depression, may explain this heterogeneity. Overall, however, we indicate an association between HL and reactive depressive symptoms, but not with respect to the scale of apathy^40^. Specifically, we found that some HL patients crossed the limit of the scale (26%), with only 8% of HC exceeding the limit. Due to the strong negative correlation between depression and quality of life in our HL patients, we assume that there is a relationship between these two variables, which deserves to be investigated.

### Comparison of patients with and without hearing aid

At baseline HL patients that refused the hearing aids were more apathetic, mostly on the emotional domain of apathy, than those who accepted hearing aids. At follow-up, patients with hearing aids worsened on abstract logical reasoning and according to an exploratory analysis without correction for multiple comparisons, they improved on long-term spatial memory and apathy as compared to patients not wearing hearing aid. We decided to explain also the exploratory results and we suggest that since HL leads to a progressive disuse of long-term memory^41^ which is compensated by a strengthening of executive functions, patients without hearing aids may have needed more executive resources, such as logical reasoning, to compensate for long-term memory loss. Moreover, the different change at follow up in abstract logical reasoning might also be explained according to the hypothesis of relocation of cognitive resources, suggesting that in HL there is a concomitant increased engagement of short-term memory and executive functions^42^.

Our patients with hearing aids showed an improvement in long-term spatial memory, that can be explained according to the hypothesis of information degradation. Indeed, since HL increases the cognitive load in the auditory processing of information thus compromising the perception phase, it is supposed that other brain networks are recruited to overcome such difficulties^43^. According to this hypothesis, the effects of hearing impairment on cognitive networks may be extensive, but also temporary and reversible, thus suggesting that a longer follow up of our patients would be needed to evaluate the long-term effect of hearing aids on cognitive abilities^43^.

Since the association between HL and cognitive deficits may have different pathogenic mechanisms, also the effects of hearing aids on cognition could have different explanations. In fact, auditory and cognitive deficits may have the same neuropathological origin, or HL could lead to a multi-morbidity cycle in different brain areas or interact with other risk factors to accelerate cognitive decline^4^. Indeed, the literature on the use of hearing aids gave mixed results. In fact, some studies found an improved cognitive performance in HL patients after aids use^11,44–46^ while other studies found that the use of long-term amplification tools had no effect on cognition and cognitive decline^45,47^. In particular, Sarant et al.^15^ assessed ninety-nine HL patients by Mini Mental State (MMSE) and CogState battery at baseline and after using hearing aids for 18 months, and found that at follow-up patients improved in speech perception in quiet environments, self-reported listening disability and quality of life. Moreover, at 18-month follow-up with hearing aids, they found either a clinically significant improvement or stability in executive function in 97.3% of participants using hearing aids, and either stability or significant improvement in global cognition, suggesting that treatment of hearing loss with hearing aids may delay cognitive decline^15^.Other previous studies, performed on very large samples but using only screening tests for cognition, found that patients with hearing aids had a better performance than patients without aids on screening tests, usually the MMSE^48^.

Taljaard et al.^11^ performed a systematic review and meta-analysis including 33 studies, and reported that hearing impairment seemed to negatively affect all cognitive domains, while any type of hearing intervention significantly improved cognition, but they also suggested that their results were not conclusive, due to differences among studies, small sample sizes, and the failure to control for premorbid and other health factors in most studies.

On the other hand, in a cross-sectional study, Saunders et al.^47^ did not find any significant differences at MOCA screening test among HL patients with hearing aids (N = 22), HL patients without aids (N = 20) and HC (N = 19). Moreover, a study on community-dwelling older adults with hearing impairment (N = 666) found no significant differences between hearing aid users and non-users in cognitive, social engagement or mental health outcomes assessed by means of MMSE, TMT, DSST, Auditory Verbal Learning Test (AVLT) and the Verbal Fluency Test (VFT) at baseline, 5 years prior to baseline and 5 and 11 years after baseline^49^.

In our study, among patients with mild HL, patients with hearing aid significantly improved on the CDT as compared to patients without hearing aids; although CDT investigated the visuo-spatial abilities, it can also be used to evaluate global cognitive state. This finding suggests that mild HL responds well to HL treatment on a short-term follow up. Indeed, the CDT is scarcely affected by the level of education, language and cultural differences than the other cognitive tests such as the MMSE and is also less affected by voice delivery; it covers a wide range of cognitive domains such as construction, visuo-spatial function, conceptualization and the executive function, and it is particularly informative since it involves the visuo-spatial function as well as the frontal inhibitory function^50^. On the other hand, among patients with moderate or severe-profound HL, abstract logical reasoning tended to worsen in patients with hearing aids as compared to patients without aids, suggesting that patients without hearing aids may have needed more executive resources, as already discussed for the whole sample, and that the location of cognitive resources may affect patients with moderate or severe profound HL more than patients with mild HL.

However, our subgroups were too small to draw firm conclusions. We can only speculate that long-term HL treatment in mild HL patients could favor a lower cognitive decline over time, but this hypothesis needs to be supported by further longitudinal studies assessing cognitive outcomes also according to the severity of HL.

The beneficial effects of hearing aid use on cognitive function are likely modest; therefore, it is necessary to confirm the effects through further investigations that involve larger sample sizes and long-term observation^22^.

The main limitation of this study is the small number of patients involved, that justified a more exploratory approach. Second, the interviewer was not blind to the diagnosis and the use of hearing aids; instead, the data was collected blindly to specific hypotheses under consideration. Third, the evaluation was conducted using a large number of neuropsychological tests, based on the Guidelines for the evaluation of dementia and age-related cognitive change, this allowed to perform a more complete analysis, but indeed increased the number of comparisons. We acknowledge that further studies on larger populations are warranted to fully explain our mixed findings on tasks exploring the same cognitive domains. Furthermore, as regards the tests, although they were chosen keeping low the hearing load, tests made for hearing impaired were not exclusively used. Finally, the brief follow up may have conditioned the reduced significance of the tests.

In conclusion, due to the prominent cognitive and social consequences, HL should be considered a social emergency. Due to the increasing prevalence of HL, associated with increasing age of the population, research into the mechanistic pathways linking HL with dementia and the potential of rehabilitative strategies to mitigate this association is warranted. The study of the cognitive profile of hearing-impaired subjects is also important, since it can contribute to a better understanding of the predictive factors of cognitive decline in the adulthood and, therefore, it is necessary to continue studying these groups of patients over time, increasing the exposure times of the subjects to the aids itself. An extensive interdisciplinary collaboration among audiologists, neurologists and neuropsychologists is deserved for future studies.

## Supplementary Information

Supplementary Information.
