# Supplementary material for: Neuropsychological profile of hearing-impaired patients and the effect of hearing aid on cognitive functions: an exploratory study
Source: Sci Rep. 2021 Apr 30;11:9384. doi: 10.1038/s41598-021-88487-y (PMC8087665; doi:10.1038/s41598-021-88487-y)
Supplement: Supplementary file 1 — Supplementary Information. [file 41598_2021_88487_MOESM1_ESM.doc]

**Supplemental Digital Content**

**Table S1: Exploratory factor analysis** and post-hoc power analysis of each factor and each test.

|  | **Factor 1** | **Factor 2** | **Factor 3** | **Achived power of test (%)** |
| --- | --- | --- | --- | --- |
| **CPM** | **.686** | .198 | - .033 | 91% |
| **MOCA- Total** | **.670** | .606 | .055 | 83% |
| **15-RAWTL- immediate recall** | .242 | **.898** | .083 | 72% |
| **15- RAWTL- delayed recall** | .107 | **.927** | .035 | 76% |
| **ROCF memory** | **.531** | .397 | - .237 | 7% |
| **ROCF copy** | **.771** | .271 | .024 | 50% |
| **CDT** | .015 | .080 | **.972** | 10% |
| **TMT-A** | **-.793** | - .090 | - .156 | 5% |
| **oDCT** | **.784** | .052 | - .067 | 15% |
| **Power of factor (%)** | 30% | 70% | 16% |  |

**Abbreviations:** 15-RAWLT Rey’s auditory 15-word learning test; BDI-II; CDT, Clock Drawing Test; CPM, Raven's Coloured Progressive Matrices; MOCA, Montreal Cognitive Assessment; oDCT, Digit Cancellation Test; ROCF, Rey-Osterrieth Complex Figure Test; TMT-A, Trail Making Test Part A.

Table S2: Description of neuropsychological tests used for the study, cognitive construct measured by the test, and impact of hearing loss.

| **Test** | **Description** | **Measured construct** | **Influenced by HL** | **Statistical effect** | **Grouping cognitive construct by factor analysis** |
| --- | --- | --- | --- | --- | --- |
| **CPM ^1^** | CPM is made up of 36 plates, divided into three series, each table is made up of an upper part that contains the stimulus figure that lacks a fragment and a lower part that contains six alternative responses. The subject is asked to indicate, among the six alternatives, the one that completes the stimulus figure. The administration has no time limits. | Mental age.  Intellectual performance.  Non-verbal intelligence.  Visuo-perceptual functions.  Visuo-spatial functions. | Not affected by HL | Yes, see Table 1, 2 and 3. | Executive/ attentional Factor |
| **MOCA ^2^** | MOCA is a rapid screening tool, which investigates eight cognitive domains such as, verbal memory immediate and deferred recall, visual-spatial skills, executive functions, attention, concentration, working memory, the language and space-time orientation. | Global cognitive state. | Affected by HL | Yes, see Table 1. | Executive/ attentional Factor |
| **ROCF-copy ^1^** | ROCF-copy is a test that involves the copy of a complex figure consisting of 18 elements. | Planning skills. Constructive praxis | Not affected by HL | No | Executive/ attentional Factor |
| **ROCF- memory ^1^** | ROCF- memory is a test that involves the copy of a complex figure consisting of 18 elements that after 15 minutes must be recalled without the aid of the stimulus. | Long-term visual-spatial memory.  Planning skills. Constructive praxis. | Not affected by HL | No. | Executive/ attentional Factor |
| **TMT-A ^1^** | TMT-A requires you to combine the numbers circled and arranged on a sheet in random order in ascending order. The administration has no time limits. The administration requires the stopwatch. | Visual-motor coordination.  Mental flexibility. Processing speed. | Not affected by HL | No. | Executive/ attentional Factor |
| **oDCT ^1^** | oDCT is composed of three numbers metrics. The subject must cross out the target numbers which are one for the first matrix, two for the second and three for the third, distinguishing them from the distractors. The administration has time limits. | Selective visual attention.  speed, detection skills.  interaction between working memory and visual-attentive processes. | Not affected by HL | No. | Executive/ attentional Factor |
| **CDT ^1^** | The examiner invites the patient to draw a clock, to insert the numbers inside and the hands that point to 11:10. | Visual-constructive functions. Visual-spatial orientation. Mental representation skills.  Planning skills. | Not affected by HL | Yes, see Table 3. | Visuo-spatial Factor |
| **15-RAWLT- immediate recall ^1^** | 15-RAWLT- immediate recall is composed by a list of 15 words. In the immediate recall test, the subject is invited to recall as many words as possible read by the examiner; the reading of the list is repeated 5 consecutive times, the examiner must record the words and the order of the recall from time to time asking the subject to also report the words previously said. | Antegrade verbal memory.  Learn verbal memory.  Long-term verbal memory.  Short-term memory. | Affected by HL | No. | Memory Factor |
| **15-RAWLT- delayed recall ^1^** | The delayed recall is foreseen after a time interval of about 15 minutes during which visuospatial tests are administered; in this case the subject is asked again to recall the greatest number of words belonging to the list read several times previously, without in this case there being a further repetition of the same (Caltagirone et al., 1995). | Antegrade verbal memory. | Affected by HL | No. | Memory Factor |

**Abbreviations**: 15-RAWLT Rey’s auditory 15-word learning test; CDT, Clock Drawing Test; CPM, Raven's Coloured Progressive Matrices; HL, hearing loss; MOCA, Montreal Cognitive Assessment; oDCT, Digit Cancellation Test; ROCF, Rey-Osterrieth Complex Figure Test; TMT-A, Trail Making Test Part A.

**Adapted by:**

^1^Kit of the Italian Neuropsychologist Barletta-Rodolfi C., Gasparini F., Ghidoni E. Italian Society of Neuropsychology Bologna (2011).

^2^Nasreddine ZS, Phillips NA, Bedirian V, Charbonneau S, Whitehead V, et al. (2005) The Montreal Cognitive Assessment, MoCA: a brief screening tool for mild cognitive impairment. J Am Geriatr Soc. 53: 695–699.

**Table S3: Description of neuropsychological tests, cognitive construct measured by the test, and impact of hearing loss derived from previous studies on HL patients.**

| **References** | **Neuropsychological test** | **Measured construct** | **Affected by HL** | **Statistical effect** |
| --- | --- | --- | --- | --- |
| **12.** | 1.DVMT ^1^ | 1. Auditory-verbal memory, memory function between the hemispheres. | 1. Affected by HL | Yes |
| **29.** | 1. MMSE ^1^ | 1. Global cognitive state. | 1. Affected by HL | No |
|  | 2. FAB ^1^ | 2. Executive function. | 2. Affected by HL | Yes |
|  | 3. TMT A ^1^ | 3. TMT-A measures visual-motor coordination, mental flexibility, processing speed. | 3. Not affected by HL | No |
|  | 4. Boston Nominating  Test ^1^ | 4. Language | 4. Not affected by HL | No |
|  | 5. ROCF ^1^ | 5. Constructive praxis. | 5. Not affected by hearing loss. | No |
|  | 6.Test Backward ^1^  Digit Span ^1^ | 6. Verbal Working Memory. | 6. Affected by HL. | No |
|  | 7. FCSRT ^1^ | 7. Explore verbal episodic memory. | 7. Affected by HL. | No |
| **31.** | 1. MMSE ^1^ | 1.Global cognitive state | 1. Affected by HL. | Yes |
|  | 2. MOCA ^2^ | 2. Global cognitive state | 2. Affected by HL. | Yes |
| **34.** | 1. MMSE ^1^ | 1. Global cognitive state | 1. Affected by HL. | No |
|  | 2. 15-RAVLT- immediate and delayed recall ^1^ | 2. Antegrade verbal memory, short-term memory | 2. Affected by HL. | Yes |
|  | 3. Digit span  forwards ^1^  Corsi backwards ^1^ | 3. Short-term memory | 3. Affected by HL. | No |
|  | 4. Digit span backwards ^1^  Corsi backwards ^1^ | 4. Working memory | 4. Affected by HL. | No |
|  | 5. MFTC ^1^ | 5. Selective and divided attention | 5. Not affected by HL. | Yes |
|  | 6. Constructional praxis ^1^ | 6. Praxis ability | 6. Not affected by HL. | No |
|  | 7. Phonological fluency task ^1^ | 7. Ability to access the verbal lexicon by phonological way, mental flexibility | 7. Not affected by HL. | No |
|  | 8. Semantic fluency task ^1^ | 8. Ability to access the verbal lexicon semantic way, mental flexibility. | 8. Not affected by HL. | No |
|  | 9. Nouns and actions naming ^1^  Auditory lexical decision ^1^  Auditory noun and actions comprehension ^1^  Auditory sentences comprehension ^1^ | 9. Language | 9. Affected by HL. | Yes |
|  | 10. CPM ^1^ | 10. Mental age, intellectual performance, non-verbal intelligence, visuo-perceptual functions,  visuo-spatial functions. | 10. Not affected by HL. | No |
|  | 11. Stroop color word test, short form ^1^ | 11. Selective attention, ability to inhibit interference, cognitive flexibility. | 11. Not affected by HL. | No |
| **46.** | 1. MOCA ^2^ | 1. Global cognitive state | 1. Affected by HL | Yes |

**Abbreviations**: 15-RAWLT Rey’s auditory 15-word learning test; CPM, Raven's Coloured Progressive Matrices; DVMT, Dichotic Auditory Memory Test; HL, hearing loss; FAB, Frontal Assessment Battery; FCSRT, Free and Cued Selective Reminding Test; MFTC, Multiple Feature Target Cancellation; MMSE, Mini‐Mental State Examination ; MOCA, Montreal Cognitive Assessment; ROCF, Rey-Osterrieth Complex Figure Test; TMT-A, Trail Making Test Part A.

**Adapted by:**

^1^ Barletta-Rodolfi et al., *Kit of the Italian Neuropsychologist*. Italian Society of Neuropsychology Bologna (2011).

^2^ Nasreddine et al. *The Montreal Cognitive Assessment, MoCA: a brief screening tool for mild cognitive impairment.* J Am Geriatr Soc. 53: 695–699(2005).
